# Exploring the influence of climatic variables on mycobiome composition and community diversity in lichens: insights from structural equation modeling analysis

**DOI:** 10.1186/s40793-023-00535-4

**Published:** 2023-10-27

**Authors:** Jiho Yang, Jung-Jae Woo, Wonyong Kim, Seung-Yoon Oh, Jae-Seoun Hur

**Affiliations:** 1 Korean Lichen Research Institute, Sunchon National University, 255 Jungang-ro, Suncheon, 57922 South Korea; 2 Department of Biology and Chemistry, Changwon National University, 20 Changwondaehak-ro, Changwon, 51140 South Korea

**Keywords:** Lichen, Endolichenic fungi, Community feature, Host haplotype, Geographical distance, Climatic variables, Isothermality

## Abstract

**Background:**

Lichens are symbiotic organisms composed of a fungus and a photosynthetic partner, which are key ecological bioindicators due to their sensitivity to environmental changes. The endolichenic fungi (ELF) living inside lichen thalli, are an important but understudied component of playing crucial ecological roles such as nutrient cycling and protection against environmental stressors. Therefore ELF community investigation is vital for fostering sustainable ecosystems and leveraging their ecological benefits. Deciphering the intricate relationships between ELF and their lichen hosts, alongside the influence of environmental factors on these communities, presents a significant challenge in pinpointing the underlying drivers of community structure and diversity.

**Results:**

Our research demonstrated that locational factors were the main drivers of the ELF community structure, rather than host haplotype. Several climatic factors affected the diversity of the ELF community and contributed to the prevalence of different types of fungal residents within the ELF community. A decrease in isothermality was associated with a greater prevalence of pathotrophic and saprotrophic fungi within the ELF community, resulting in an overall increase in community diversity. By conducting a structural equation modeling analysis, we identified a robust link between climatic variables, fungal trophic mode abundance, and the species diversity of the ELF community.

**Conclusion:**

This study’s discoveries emphasize the significance of examining climate-related factors when investigating ELF’s structure and function. The connection between fungi and climate is intricate and complex, and can be influenced by various other factors. Investigating the potential for ELF to adapt to changing climatic conditions, as well as the potential effects of changes in ELF communities on lichen function, would be valuable research areas. We anticipate that our research results will establish a basis for numerous future ELF research projects and have a significant impact on the field.

**Supplementary Information:**

The online version contains supplementary material available at 10.1186/s40793-023-00535-4.

## Introduction

Lichens are commonly recognized as a symbiotic association between two partners, usually green alga and/or a cyanobacterium, however they are actually a community of microorganisms [1]. The fungal partner, which forms the structural framework of the lichen thallus, provides a protected habitat for the photobiont and exchanges nutrients with it [2]. Lichens can colonize a wide range of terrestrial habitats, from deserts to rainforests, and are used as important bioindicators of environmental change due to their sensitivity to air pollution and climate variability [3].

Endolichenic fungi (ELF) representing fungal residents inside lichen thallus without causing disease symptoms are diverse and abundant [4], and have been found in almost all lichen species examined to date [5]. They belong to various taxonomic groups, and many have been still undescribed [6], Therefore, they represent an understudied component of fungal diversity, and are raising great interest in the ability to produce secondary metabolites with a wide range of biological activities [7]. ELF were considered to play important ecological roles in their host, as in nutrient cycling, carbon fixation, and protection against environmental stressors such as UV radiation and desiccation [5]. Therefore, studying and understanding ELF communities is not only important for gaining knowledge about their species diversity and distribution, but also for uncovering their ecological roles and interactions. This knowledge can help to promote sustainable ecosystems and ecological benefits.

Several factors including lichen host, environmental conditions, and interactions with other microorganisms can affect the structure of ELF community [5, 8]. Vertical transmission of microbiome: from parent thalli to offspring thalli [9] can have implications for the evolution and diversification of ELF. On the other hands, ELF may be adapted to the specific environmental conditions within their host lichens, which can include factors such as temperature, humidity, and nutrient availability. Evidence supporting this speculation includes culture-based studies conducted on a continental scale [10] and NGS data indicating the presence of several lichenicolous fungi in a single locality regardless of host species [11]. The ELF may interact with other microorganisms that co-occur within lichens, such as bacteria or other functionally distinct fungi. These interactions can affect the growth and distribution of ELF. Despite recent advances in our understanding of ELF communities, there is still a lack of data on the factors that strongly influence the formation of these communities. The complex interactions between ELF and their host lichens, as well as the environmental factors that shape these communities, make it difficult to identify specific drivers of community composition and diversity.

To address the knowledge gap on ELF and their communities, we designed a research that aims to explore the diversity and composition of ELF. To investigate whether ELF are vertically or horizontally transmitted, we targeted the lichen species *Parmotrema tinctorum* that is distributed evenly across different regions [12] as our study subjects. By sampling samples of this species from different locations and analyzing the ELF communities within them, we can determine if the presence and composition of ELF are consistent across host lichen populations or if they vary based on the geographic location. Given the potential for significant variation in ELF community based on location, we set out to investigate the climatic factors that underpin this diversity. Additionally, we aimed to track the trophic mode of members within the ELF community to determine how each resident contributes to the diversity of the community.

## Materials and methods

### Lichen sampling

**Fig. 1:**
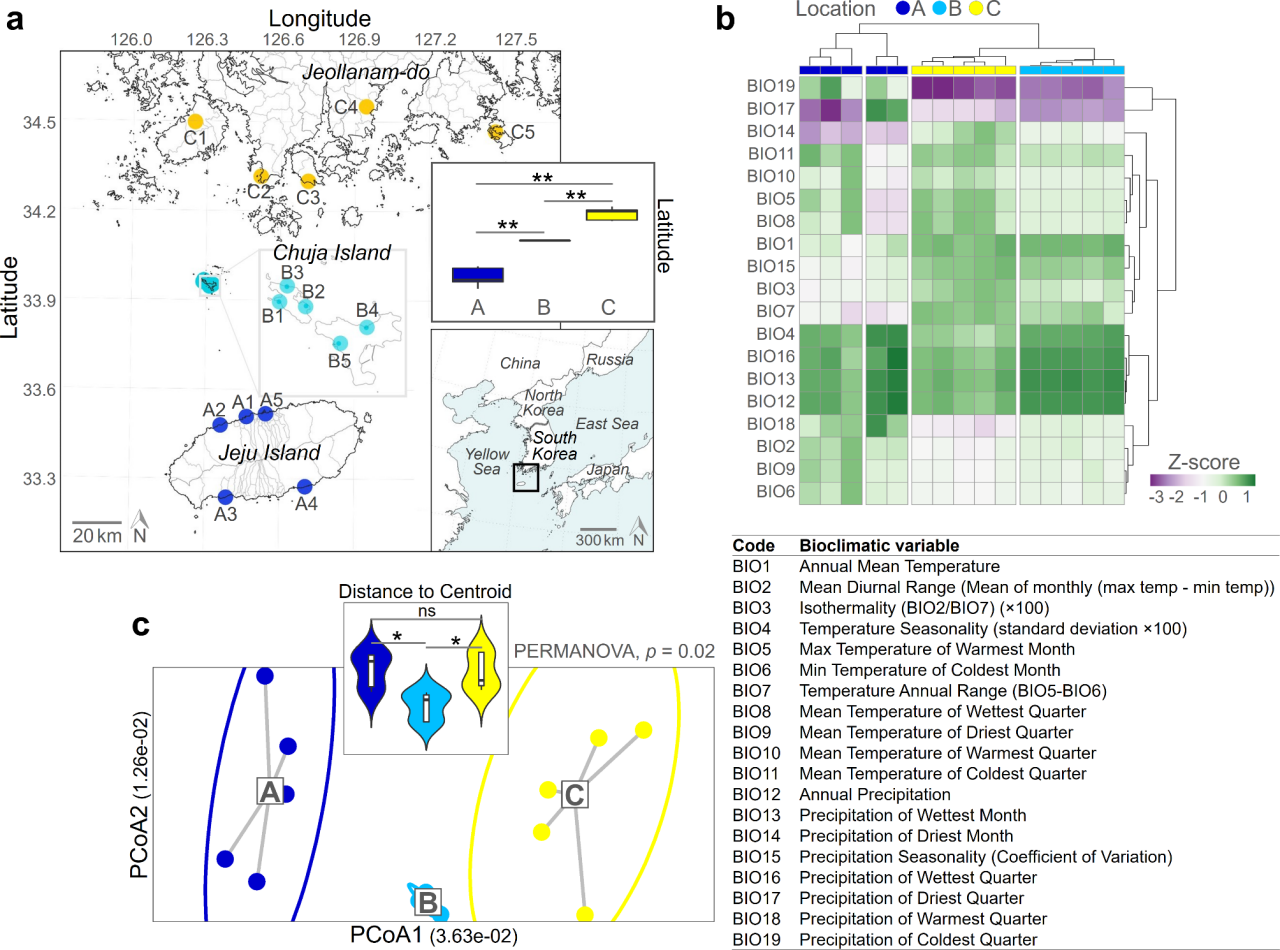
Lichen thalli of the same species, distributed across various locations with different climatic variables, were collected. (**a**) Collection map of host lichens. A total of 150 epiphytic *Parmotrema tinctorum* were collected, with ten samples from each of the five sub-sites at three geographically distinct areas with varying latitudes. The mapping was visualized in QGIS v.3.6. (**b**) Hierarchical heatmap and (**c**) principal coordinate analysis ordination revealed statistically significant differences in the climatic factors across the three regions. * *p* < 0.05, ns *p* ≥ 0.05

We collected 150 lichen samples at three locations: Jeju Island, Chuja Island, and Jeollanam-do separated by the South Sea located off the southwestern part of South Korea in 2019 (Fig. 1a). While administratively belonging to Jeju Province, Chuja Island is geographically located between Jeju Island and the mainland of the Korean peninsula intermediately. We collected 10 thalli of epiphytic lichen *Parmotrema tinctorum* from each of five sub-sites at each location (in total, 150 lichens in 15 sub-sites) (Supplementary file 1). Morphological identification of each lichen sample was performed in the field, followed by molecular identification based on internal transcribed spacer (ITS) region in the laboratory. The lichen thallus, obtained during this study, was cut into 1 cm^2^ piece and its surface residues were eliminated using a syringe tip and by washing them with running tap water. Following this, the surface of the lichen thalli was sterilized according to Yang’s protocol [13] using a solution of 70% ethanol and 0.4% sodium hypochlorite for 90 s.

### Molecular and bioinformatics analyses

The surface-sterilized lichen segments were homogenized by lysing beads and their DNA was extracted using a PowerSoil DNA isolation kit (QIAGEN, CA, USA). After molecular identification of *P. tinctorum*, we conducted polymerase chain reaction (PCR) of the fungal ITS1 region [14] using primers ITS1F and ITS2 [15] ligated to Illumina sequencing adaptors. We performed PCR three times for each sample using the AccuPower PCR PreMix kit (Bioneer, Daejeon, South Korea) based on the following condition: 94 ℃ for 5 min, 25 cycles of 94 ℃ for 30 s, 55 ℃ for 30 s, and 72 ℃ for 40 s with a final extension at 72 ℃ for 10 min. We evaluated the quality of PCR products on agarose gel and purified using the Expin PCR SV kit (GeneAll, Seoul, South Korea). Second PCR for barcoding was conducted to attach multiple index delimiters as recommended by the Nextera XT Index kit protocol (Illumina, CA, USA). Amplicon concentration was measured using the NanoDrop2000 (Thermo Fisher Scientific, MA, USA) and PCR products were pooled in equal molecular quantity.

Amplicon library sequencing was performed using an Illumina Miseq platform by Macrogen (Seoul, South Korea). Raw sequences were processed using QIIME2 [16] v.2021.4 by demultiplexing and denoising reads following the DADA2 pipeline [17]. Taxonomic assignment was performed according to the Naïve Bayesian classifier guideline [18] using the UNITE fungi 99% OTU database [19]. A phylogenetic analysis was conducted using the q2-alignment plugin based on RAxML [20]. Sequencing data were deposited in National Center for Biotechnology Information (NCBI), Sequence Read Archive under accession number PRJNA955390. The amplicon sequence variant (ASV) [21] table was imported from QIIME2 to R using “qiime2R” [22]. The sequences of host lichen were filtered out and the ASV table were rarefied to 2,000 sequences per lichen host using “phyloseq” [23]. In this process, 30 Location A samples, 8 Location B samples, and 22 Location C samples were excluded. Following analyses were based on the rarefied ASV table.

### Characterizing host haplotypes and environmental parameters in sampling locations

To determine which factor - host or location - had a great influence on ELF community structure, we conducted following genetic analyses of the host lichen *P. tinctorum*. ITS region of host lichen was used in phylogenetic analysis with other *Parmotrema* species sequences deposited in NCBI database. Phylogeny tree was established using MEGA v.7 [24] based on maximum-likelihood [25] based on Jukes-Cantor model [26], which recorded the lowest Bayesian information criterion [27] in model test with 1,000 replicated bootstrap value and visualized in iTOL [28]. We calculated the genetic variations of *P. tinctorum* populations with the number of variation sites, nucleotide diversity, number of haplotypes, and haplotype diversity using DnaSP v.6 [29]. We generated haplotype network using PopART v.1.7 [30] based on the method of Templeton, Crandall and Sing [31]. Discriminant analysis of the principal components (DAPC) was conducted to identify haplotype clusters based on k-means clustering [32]. We calculated phylogenetic distance using R package “adephylo” with “distRoot” function [33]. We calculated the distance to the root using two different approaches: maximum-likelihood and Bayesian inference [34]. The Bayesian tree was constructed using the programs BEAUti, BEAST, and TreeAnnotator [35]. To check locational effect on the ELF community, we obtained bioclimatic data (BIO01 to BIO19) for the collection sites by utilizing the WorldClim [36] database based on GPS coordinates.

### Statistics and visualizations

All following analyses were performed using software R v.3.5.3 [37]. For the statistical comparison, we conducted the Shapiro test [38] to check data normality at the first. Since the normality assumption was violated, we used the non-parametric test using the Mann–Whitney U test [39] in pairwise comparison and the Kruskal-Wallis test [40] followed by the Bonferroni correction [41] in multiple comparison. To test statistical difference of bioclimatic values of collection sites, we conducted principal coordinate analysis (PCoA) [42] with PERMANOVA test [43] using “vegan” package [44] and hierarchical clustering with “pheatmap” package [45]. The collection sites were marked with different colors for each region and visualized in Quantum Geographic Information System v.3.6 (http://qgis.osgeo.org/). All plot visualizations were performed using “ggplot2” and “ggpubr [46]” packages. Fungal community similarity was calculated based on Bray-Curtis distance [47] with “vegan” package. Using Bray-Curtis distance, non-metric multidimensional scaling (NMDS) was visualized using “phyloseq” and “vegan” packages. Our analysis for investigating relationship between the ELF community diversity and the bioclimatic variables was conducted using the “ggpairs” package. Different abundance of fungal residents across locations was identified by chi-squared test and visualized with volcano plot (https://github.com/kevinblighe). Simple and multiple regression analyses were performed to investigate the correlation between the ELF community and environmental factors. We traced the fungal trophic mode by referencing previous reports [11, 48–54] and utilizing the FUNGuild database [53]. Structural equation modeling (SEM) is a statistical method used to analyze the relationships between latent variables [55]. Our analysis involved setting up three latent variables, namely bioclimatic variables, community residents, and community diversity, and evaluating their explanatory power with statistical significance. SEM analysis was conducted using “lavaan” [56], “lavaanPlot”, “dplyr”, “tidyr”, “knitr”, and “mvnormalTest” packages.

## Results

### Location and haplotype information of host lichen

We collected 150 samples of *P. tinctorum* at three different locations: Jeju Island (represented as Location A), Chuja Island (Location B), and Jeollanam-do (Location C) (see Materials and Methods, Fig. 1a). The three regions are geographically separated by the South Sea and differ in their latitudes. The three locations also differed significantly in terms of their climate variables (PERMANOVA, *p* = 0.02) e.g., BIO19: precipitation of coldest quarter was the highest in Location C followed by Locations B and A (Fig. 1b and c).

**Fig. 2:**
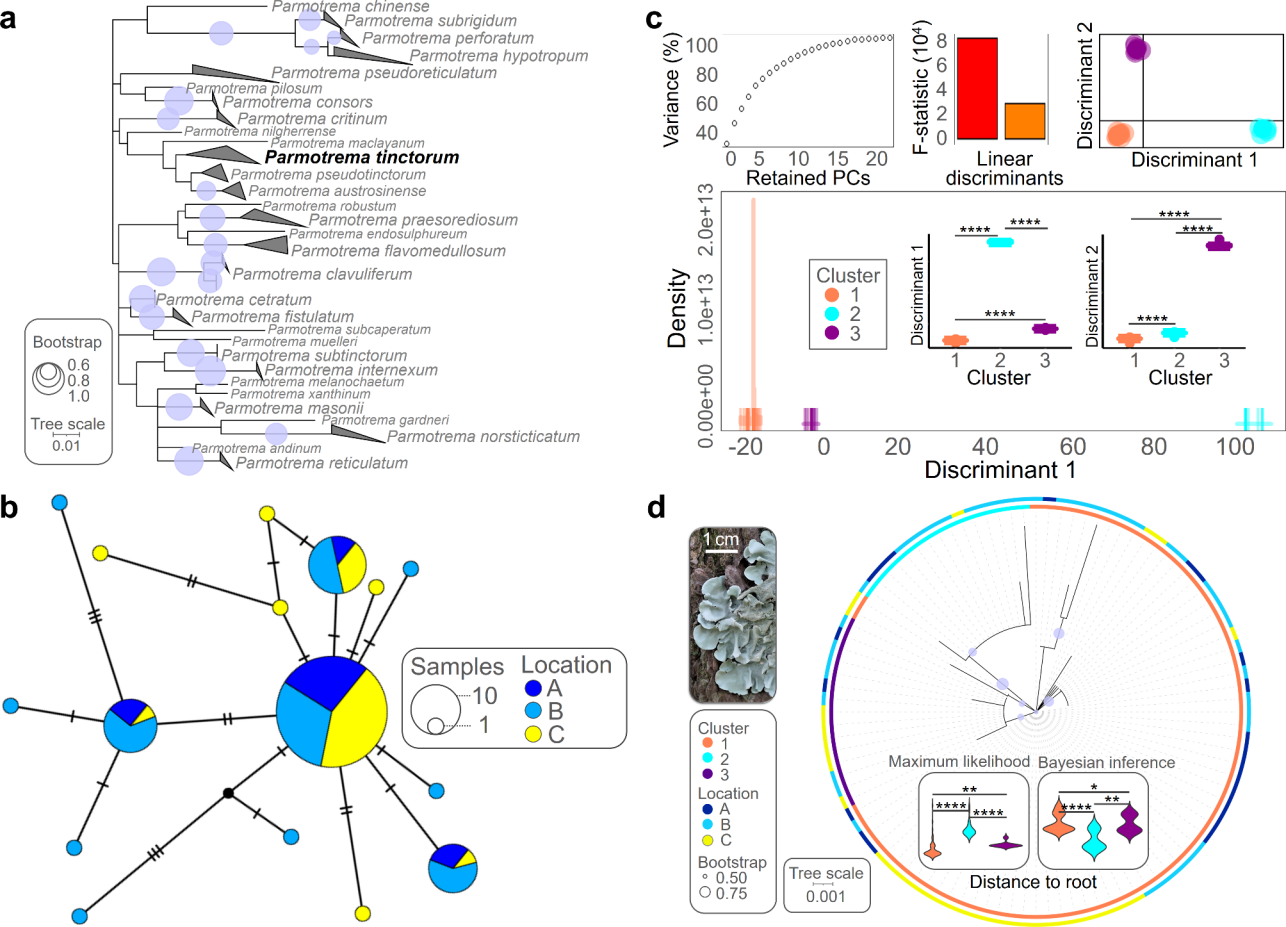
Clustering of the collected lichens into three haplotype groups was determined by sequence variations. (**a**) Phylogeny of *Parmotrema* using sole internal transcribed spacer (ITS) locus indicated that ITS gene appeared to be appropriate for further haplotype analysis. (**b**) The collected lichens were determined to comprise 16 haplotypes based on genetic diversity. The method of Templeton, Crandall and Sing was employed to generate the haplotype network, which was visualized using PopART v.1.7. (**c**) A grouping of the 16 haplotypes into three clusters was achieved through discriminant analysis of the principal components (PC). Clustering was performed by utilizing the two PCs with the highest explanatory power, despite obtaining 20 PCs to confirm sequence variation among haplotypes. (**d**) Based on the phylogenetic analysis of the lichen species used in the study, the haplotype clusters were randomly distributed in the locations. The Cluster 2 demonstrated the most sequence variations from the common ancestor. The phylogenetic trees in this figure were constructed by MEGA v7.0 based on maximum-likelihood method with 1,000 bootstrap replications and visualized through iTOL

The ITS region of lichen *Parmotrema* provided enough information for molecular species identification through sole locus itself and this suggested that the gene locus can be conveniently employed for haplotype analysis (Fig. 2a). Upon analyzing the genetic variation, we found that the 150 collected *P. tinctorum* exhibited sixteen distinct haplotypes. (Fig. 2b). To investigate haplotype cluster, we conducted discriminant analysis of the principal components (Fig. 2c). We performed haplotype clustering using the two discriminants, which had adequate statistical explanatory power. The results of the analysis demonstrated that the sixteen haplotypes were divided into three clusters (Supplementary file 1). The phylogeny of host lichens revealed that the three haplotype clusters were arbitrarily dispersed among the three locations (Fig. 2d). It’s worth noting that there was a significant disparity in the distance to the root when assessed through the maximum-likelihood method compared to the Bayesian inference tree. Furthermore, it’s important to highlight that the distances between the three haplotype clusters displayed statistical significance in both of these approaches.

### Geographical factor had a more significant impact on the ELF community than the host’s haplotype

**Fig. 3:**
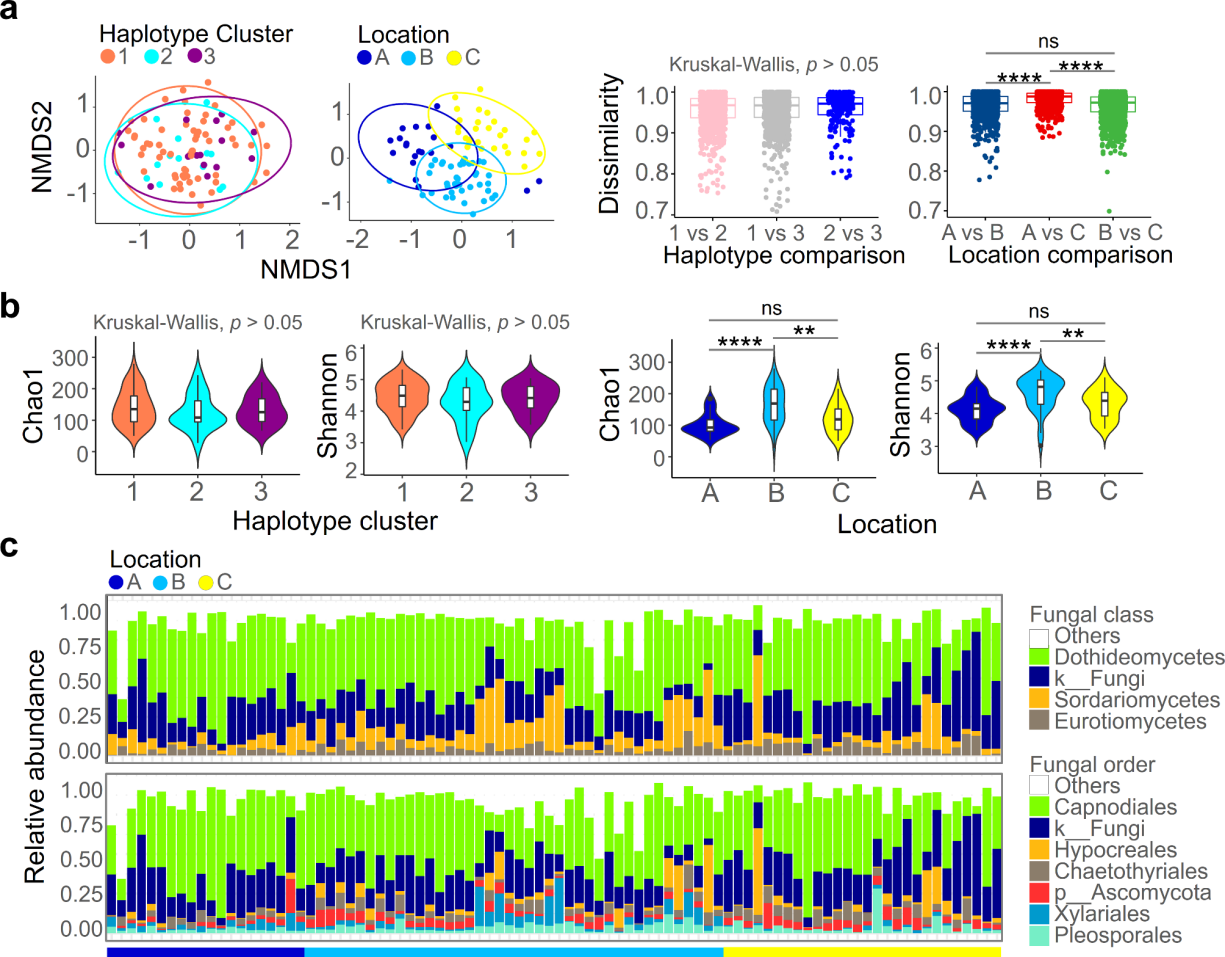
Structure of the ELF community was found to be more influenced by geographic factors than the haplotype of the host. (**a**) Non-metric multidimensional scaling and comparison of community dissimilarity suggested that locational difference had a greater impact than host haplotype on the formation of the ELF community. The community dissimilarity was calculated based on Bray-Curtis distance. (**b**) Alpha diversity indices (Chao1 richness and Shannon’s diversity) of the ELF community. (**c**) Taxonomic composition of the ELF community was visualized in fungal class and order levels. **** *p* < 0.001, ** *p* < 0.01, ns *p* ≥ 0.05

Fungal community similarity analyses revealed that the structure of the ELF community was greatly influenced by location factors rather than the host’s haplotype (Fig. 3a). Of note, the Bray-Curtis distance analysis of the ELF community indicated that the difference in community structure between Locations A and C was the greatest. Taken together, these findings suggest that the ELF community located in Location B exhibited intermediate features between the fungal communities found in Locations A and C, resembling a locational intermediate.

We also observed differences in the alpha diversity indices of the ELF communities in relation to the location variations (Kruskal-Wallis, *p* < 0.01) rather than the host haplotype clusters (Fig. 3b). The ELF community located in B showed the highest species richness (Chao1) and diversity (Shannon). To examine the differences in the internal features of the ELF communities across locations, we examined the taxonomic composition of the fungal communities (Fig. 3c). Consistent with previous studies [11], members of the fungal order Capnodiales were found to be the most dominant residents within the ELF communities. The dominance of Capnodiales was less pronounced in Location B and Sordariomycetes fungi showed a higher frequency in Location B compared to Locations A and C.

### Several climatic factors influenced the diversity of the ELF community

**Fig. 4:**
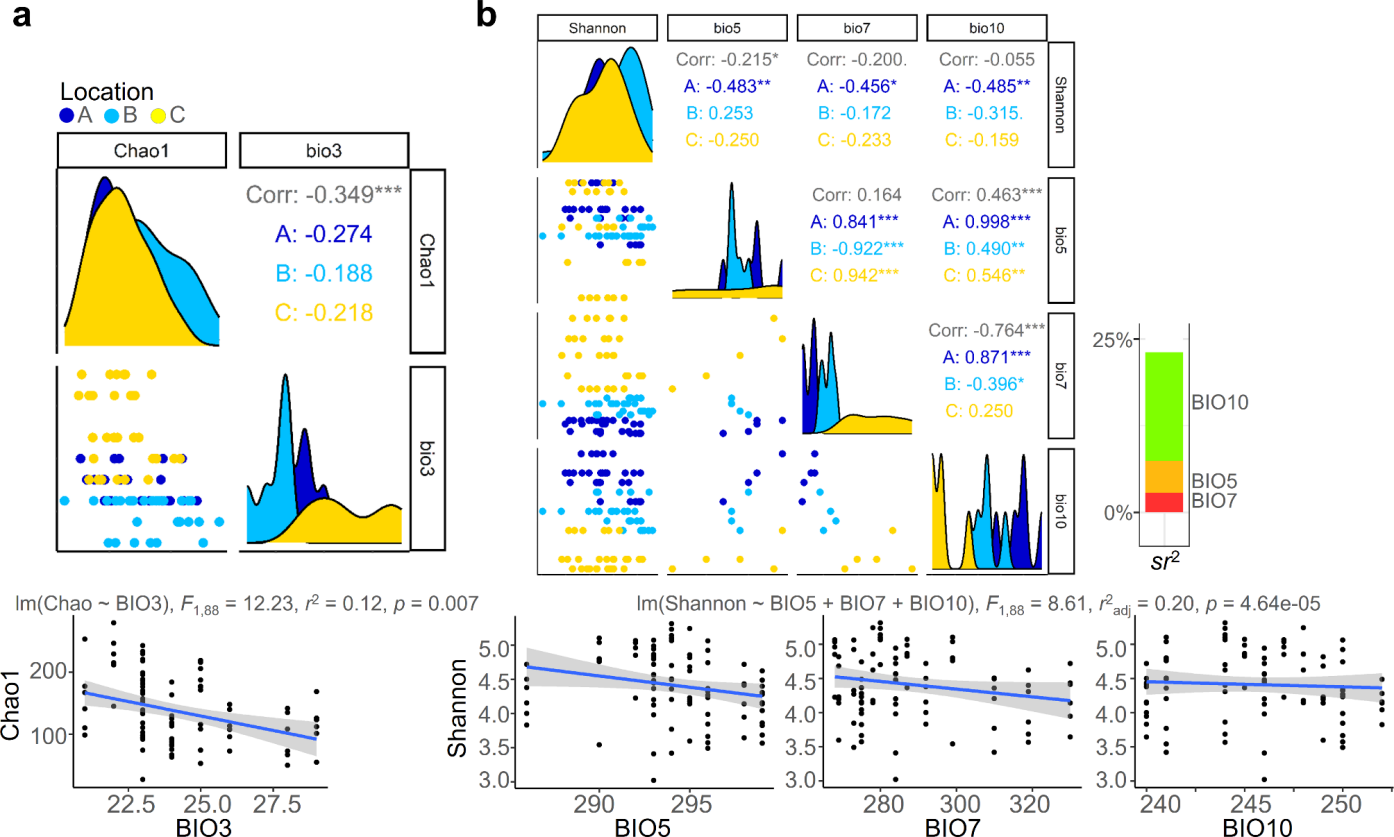
Several bioclimatic factors played a role in shaping the diversity of the ELF community. (**a**) Investigating the relationship between fungal species richness (Chao1) and isothermality (BIO3). (**b**) Analyzing the correlation of fungal species diversity (Shannon), mean temperature of warmest quarter (BIO5), max temperature of warmest month (BIO7), and temperature annual range (BIO10). The topmost distribution and scatter plots displayed the data distribution for each variable and Spearman correlation value between variables. The right-side stacked bar plot illustrated the semi-partial correlations of each variable. By performing multiple regression analysis with statistical significance and confirming multicollinearity with variance inflation factor calculation, we extracted climate data that are significantly associated with fungal diversity. *** *p* < 0.005, ** *p* < 0.01, * *p* < 0.05

As we confirmed the phenomenon of the three regions having different bioclimatic variable values, we investigated the impact of climatic factors on the diversity of the ELF community. Multiple regression analysis revealed that BIO3, BIO4, BIO5, BIO7, BIO10, BIO11, BIO12, and BIO13 had significant positive or negative relationship to fungal richness (Table 1). Among significant variables, we checked multicollinearity with variance inflation factor (VIF). The variables other than BIO3 and BIO5 were excluded from the further analysis because their VIF values exceeded 20, indicating the presence of multicollinearity. The multiple regression analysis resumed showed that BIO3 (isothermality) was the most significant climatic factor negatively affecting fungal richness in the ELF communities (*F*_1,88_ = 12.23, *r*^2^ = 0.12, *p* = 0.007) (Fig. 4a). We explored the climatic factors influencing fungal diversity (Shannon diversity index, Table 2) using the same analysis method as above, and the results revealed that variables BIO5, BIO7, and BIO10 were significantly correlated with diversity of the ELF communities (*F*_1,88_ = 8.61, *r*^2^_adj_ = 0.20, *p* = 4.64e-05) (Fig. 4a). These results suggested that isothermality, maximum temperature of the warmest month, temperature annual range, and mean temperature of the warmest quarter had a negative impact on the fungal diversity of the ELF communities.

**Table 1:** Multiple regression analysis result between fungal richness and climatic variables

	Coefficient	Std. Error	t value	*p* (whole)	*p* (1st bwd.)	VIF
(Intercept)	46.77	3242	0.01	0.99		
bio1	11.92	20.96	0.57	0.57		
bio2	-1.52	9.36	-0.16	0.87		
**bio3**	**115.58**	**37.76**	**3.06**	0.003**	0.005***	**4.6**
bio4	3.47	0.95	3.66	0.05		
bio5	61.29	14.57	4.21	0.011**	0.04*	21.03
bio6	-1.89	6.51	-0.29	0.77		
bio7	-63.11	15.47	-4.08	0.009**	0.014**	21.61
bio8	-4.24	7.08	-0.6	0.55		
bio9	0.11	0.92	0.12	0.91		
bio10	-134.32	34.29	-3.92	0.006**	0.023**	28.17
bio11	45.37	16.49	2.75	0.07		
bio12	3.32	1.53	2.17	0.05		
bio13	-15.75	7.63	-2.06	0.05		
bio14	NA	NA	NA	NA		
bio15	NA	NA	NA	NA		
bio16	NA	NA	NA	NA		
bio17	NA	NA	NA	NA		
bio18	NA	NA	NA	NA		
bio19	NA	NA	NA	NA		

We conducted the multiple regression analysis based on back selection: statistically significant variables were chosen, and confirmed as independent variables after multicollinearity test with variance inflation factor. 1st bwd, *p* value achieved after the first backward selection; VIF, variance inflation factor

**Table 2:** Multiple regression analysis result between fungal diversity and climatic variables

	Coefficient	Std. Error	t value	*p* (whole)	*p* (1st bwd.)	VIF
(Intercept)	4.31	31.99	0.14	0.89		
bio1	0	0.21	0	1		
bio2	-0.05	0.09	-0.53	0.6		
bio3	0.67	0.37	1.79	0.08		
bio4	0.02	0.01	2.57	0.012*	0.33	18.51
**bio5**	**0.49**	**0.14**	**3.42**	0.001**	0.002**	**6.99**
bio6	0.01	0.06	0.08	0.93		
**bio7**	**-0.42**	**0.15**	**-2.77**	0.008**	0.0001***	**19.82**
bio8	-0.04	0.07	-0.62	0.54		
bio9	0	0.01	0.42	0.67		
**bio10**	**-0.95**	**0.34**	**-2.81**	0.006**	0.0004***	**17.76**
bio11	0.32	0.16	1.97	0.05		
bio12	0.03	0.02	1.68	0.13		
bio13	-0.11	0.08	-1.52	0.13		
bio14	NA	NA	NA	NA		
bio15	NA	NA	NA	NA		
bio16	NA	NA	NA	NA		
bio17	NA	NA	NA	NA		
bio18	NA	NA	NA	NA		
bio19	NA	NA	NA	NA		

The multiple regression analysis were performed as same method of above analysis (see Table 1)

### The climatic factors played a role in shaping the prevalence of different types of fungal residents within the ELF community

**Fig. 5:**
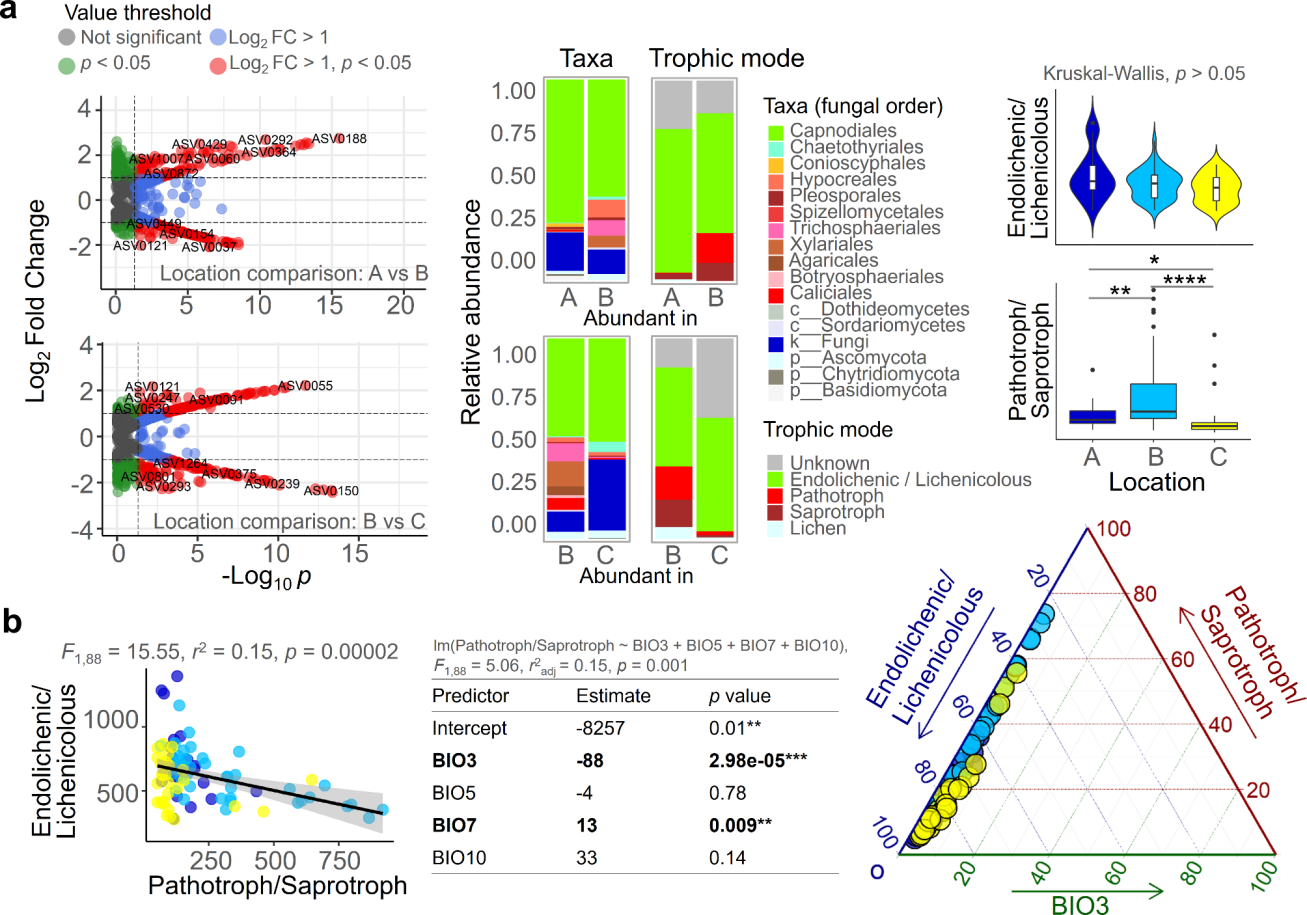
The prevalence of different types of fungal residents within the ELF community was shaped by climatic factors. (**a**) Differentially abundant fungal residents across locations were identified based on chi-squared test and visualized in volcano plot. The stacked bar plot, violin plot, and box plot demonstrated that the fungal composition of the ELF community was distinct among the three regions, with differences in both the taxonomic and ecological profiles. (**b**) Regression analyses and ternary plot showed a significant correlation of isothermality (BIO3), amount of Endolichenic/Lichenicolous and Pathotroph/Saprotroph sequence reads. **** *p* < 0.001, *** *p* < 0.005, ** *p* < 0.01, * *p* < 0.05

The results of the chi-squared test revealed differences in the abundance of ELF across locations (Fig. 5a). After tracking the taxonomic composition of the fungi with differential frequencies, it was found that the fungi belonging to the order Capnodiales were dominant in both Locations A and C. Although Capnodiales fungi were also frequent in Location B, their dominance was relatively lower than that in other locations, while fungi belonging to the class Sordariomycetes were more frequent in Location B than in other locations. According to an analysis of the trophic mode of fungi based on their taxa, endolichenic/lichenicolous fungi were more abundant in Locations A and C, while pathotrophs/saprotrophs were more commonly observed in Location B compared to other areas. The fungal populations in these two groups displayed an inverse relationship in terms of their occurrence (*F*_1,88_ = 15.55, *r*^2^ = 0.15, *p* = 0.00002) (Fig. 5b). To explore the relationship between the abundance of the pathotroph/saprotroph groups and climatic factors, we conducted a multiple regression analysis. Among the four factors that demonstrated a significant correlation with fungal diversity, BIO3 was identified as the most significant predictor of the frequency of the pathotroph/saprotroph group (estimate = -88, *p* = 2.98e-05). To summarize, a downward trend in BIO3 corresponded to a rise in the abundance of the pathotroph/saprotroph group and a decline in the quantity of the endolichenic/lichenicolous group.

### The relationship between climatic variables, fungal trophic mode abundance, and species diversity of the ELF community was explicated through the modeling approach

**Fig. 6:**
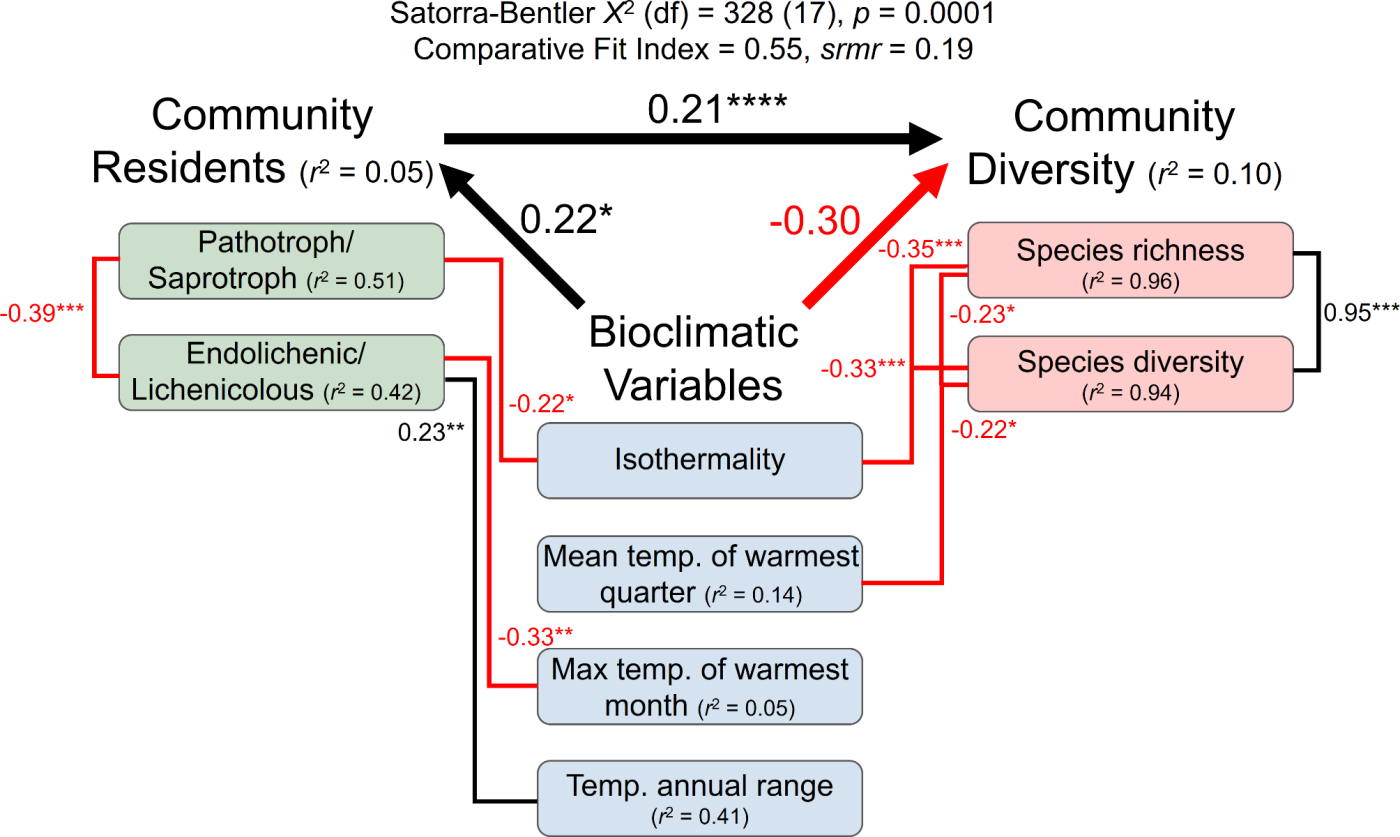
Structural Equation Modeling (SEM) provided an analytical framework for understanding the relationships and causal pathways between variables bioclimatic variable, residents and diversity of the ELF community. We defined bioclimatic variables, resident type and diversity of the ELF community as latent variables and tested the correlations between them for both explanatory power (coefficient) and statistical significance (*p* value) using SEM analysis. In the diagram, the thick arrows indicated the coefficients between the latent variables, and the lines connecting the numeric variables represent the strength of the Spearman correlations. The color scheme in the diagram was chosen such that positive correlations were denoted by black lines and negative correlations by red lines

To integrate the regression equations analyzed above and provide an explanation of correlation of fungal community and climatic factors, we constructed a structure equation model (comparative fit index = 0.55, *srmr* = 0.19) (Fig. 6). Our findings suggest that the bioclimatic variables were associated with a reduction in the diversity of the elf community (coefficient = -0.30, *p* > 0.05) and changes in its member proportions (coefficient = 0.22, *p* < 0.05): the decrease in climatic variables isothermality (Spearman correlation = -0.22, *p* < 0.05) and max temperature of warmest month (Spearman correlation = -0.33, *p* < 0.01) was found to be related to an increase in pathotroph/saprotroph group, while the increase in temperature annual range was associated with an increase in endolichenic/lichenicolous members within the community (Spearman correlation = 0.23, *p* < 0.01). The altered demographic makeup of the ELF community membership played a crucial role in shaping the community diversity (coefficient = 0.21, *p* < 0.0001). We observed a correlation between increasing pathotroph/saprotroph and greater levels of both species richness and diversity within the ELF community. Taken together, a reduction in several climatic condition leaded to an increase in pathotroph and saprotroph colonization and lower abundance of endolichenic or lichenicolous group. As a consequence, the species diversity within the ELF community was enhanced.

## Discussion

With the increasing importance of microbiome research, there has been growing interest in ELF, but the factors that determine their community structure have remained elusive. Previously, it has been believed that ELF communities are formed horizontally and are influenced by environmental factors. Nonetheless, there has been not sufficient mathematical evidences and research trials, such as a combination of haplotype clustering of homogenous lichen hosts and various climatic data. To elucidate whether ELF community formation occurs vertically or horizontally, we collected same species of host lichen from different locations and investigated the relative impact of host and environmental factors on the ELF community structure.

The results of this study provided an evidence that the structure of the ELF community was significantly influenced by climate factors. Our analyses revealed that the presence of certain fungal groups within the lichen thalli varied in response to several climatic variables. Specifically, we observed that the abundance of pathotroph and saprotroph increased with decreasing isothermality. Other climatic factors including temperature annual range, mean and maximum temperature of warmest month also were found to affect fungal diversity on the ELF community, nonetheless, isothermality was considered to most powerful predictor shaping the structure of the ELF community. These findings are consistent with previous research indicating that ELF are highly adapted to their environment [10]. Fungal residents inside lichen thallus may have evolved specialized strategies to cope with different climatic conditions. For example, some fungal species may produce pigments to protect against high levels of UV radiation [57], while others may produce enzymes to break down complex organic matter in arid environments [58]. The observed changes in the structure of ELF can therefore be seen as adaptations to the specific environmental conditions present in the lichen thalli.

Isothermality in this study refers to the degree to which temperatures remain stable over time, with minimal seasonal variation, and high isothermality value represents large diurnal temperature range. Several fungi are known to be sensitive to changes in temperature [59, 60], and as such, isothermality may play an important role in shaping fungal community structure and function. Isothermality may therefore benefit fungal communities by providing a stable, predictable environment in which to grow and reproduce. This is particularly true for fungal symbionts that live in association with plants or other organisms, where temperature stability can help to maintain the delicate balance of the symbiotic relationship [61, 62]. At the same time, isothermality may also limit the range of fungal communities that can exist in a given environment.

It is a remarkable phenomenon how fungi occupying distinct ecological niches: endolichenic/lichenicolous and pathotroph/saprotroph clusters within the lichen exhibited different responses to fluctuations in climate. The Capnodiales and Chaetothyriales known as endolichenic/lichenicolous group [11] have one of the most distinctive features: the production of dark pigments, often in the form of melanin or related compounds [63]. These pigments can help to protect the fungus from environmental stressors such as UV radiation, and may also play a role in their ability to colonize a wide range of habitats, from soil and decaying plant material to living plant tissues and even insects [64–66]. Generally speaking, highly melanized fungi tend to be slow-growing, and may require longer incubation times than non-melanized species to reach maturity [67]. Furthermore, melanized fungi were reported to have a greater capacity to withstand fluctuations in temperature compared to non-melanized fungi [68].

Xylariales that act as opportunistic pathogens and Agaricales that are primarily saprotrophs are generally not considered to be highly melanized [69, 70]. While some species within these groups may produce small amounts of melanin, it is not a defining characteristic of these fungi. Instead, Xylariales are known for their ability to colonize a wide range of substrates and their development of specialized structures, such as stromata and perithecia [71].

## Conclusion

The findings of this study highlight the importance of considering climate factors when studying the structure and function of ELF. Regarding the effect of host haplotype on the mycobiome inside lichen thalli, our results suggest that this factor may have a limited influence. Our findings indicate that highly melanized fungal residents exhibit a remarkable ability to withstand a wide range of temperatures, highlighting the role of melanin in thermal resilience. The relationship between fungi and climatic condition is complex and multifaceted, and may depend on a range of other factors such as soil moisture, nutrient availability, and thermal regimes. Further research is needed to explore the specific mechanisms by which climate factors affect fungal structure, and to develop strategies for mitigating the potential impacts of climate change on these important symbiotic organisms. In particular, studies investigating the potential for ELF adaptation to changing climatic conditions, and the potential for shifts in ELF communities to affect lichen functioning, would be valuable areas of future research. We anticipate that our research findings will lay the groundwork for various future ELF studies and contribute significantly to the field.

### Electronic supplementary material

Below is the link to the electronic supplementary material.

Supplementary Material 1

## Data Availability

All data are accessible on Sequence Read Archive (PRJNA955390), NCBI.

## Abbreviations

ASV: Amplicon sequence variant
DAPC: Discriminant analysis of principal components
ELF: Endolichenic fungi
ITS: Internal transcribed spacer
NCBI: National Center for Biotechnology Information
NMDS: Non-metric multidimensional scaling
PCoA: Principal co-ordinates analysis
PCR: Polymer chain reaction
SEM: Structural equation modeling

## References

[1] Honegger R (1998). The lichen symbiosis—what is so spectacular about it?. The Lichenologist.

[2] Honegger R (1991). Functional aspects of the lichen symbiosis. Annu Rev Plant Biol.

[3] Hawksworth DL (1990). The long-term effects of air pollutants on lichen communities in Europe and North America. The Earth in transition: patterns and processes of biotic impoverishment.

[4] Petrini O, Hake U, Dreyfuss MM (1990). An analysis of fungal communities isolated from fruticose lichens. Mycologia.

[5] Suryanarayanan TS, Thirunavukkarasu N (2017). Endolichenic fungi: the lesser known fungal associates of lichens. Mycology.

[6] Kellogg JJ, Raja HA (2017). Endolichenic fungi: a new source of rich bioactive secondary metabolites on the horizon. Phytochem Rev.

[7] Agrawal S, Deshmukh SK, Reddy MS, Prasad R, Goel M (2020). Endolichenic fungi: a hidden source of bioactive metabolites. South Afr J Bot.

[8] U’Ren JM, Lutzoni F, Miadlikowska J, Arnold AE (2010). Community analysis reveals close affinities between endophytic and endolichenic fungi in mosses and lichens. Microb Ecol.

[9] TUNJIĆ M (2013). Vertical and horizontal gene transfer in lichens. Periodicum Biologorum.

[10] U’Ren JM, Lutzoni F, Miadlikowska J, Laetsch AD, Arnold AE (2012). Host and geographic structure of endophytic and endolichenic fungi at a continental scale. Am J Bot.

[11] Fernández-Mendoza F, Fleischhacker A, Kopun T, Grube M, Muggia L (2017). ITS 1 metabarcoding highlights low specificity of lichen mycobiomes at a local scale. Mol Ecol.

[12] Jayalal U, Divakar PK, Joshi S, Oh S-O, Koh YJ, Hur J-S (2013). The Lichen Genus Parmotrema in South Korea. Mycobiology.

[13] Yang JH, Oh S-Y, Kim W, Woo J-J, Kim H, Hur J-S (2021). Effect of isolation conditions on diversity of endolichenic fungal communities from a foliose lichen, Parmotrema Tinctorum. J Fungi.

[14] Iwen PC, Hinrichs SH, Rupp ME (2002). Utilization of the internal transcribed spacer regions as molecular targets to detect and identify human fungal pathogens. Med Mycol.

[15] Larena I, Salazar O, González V, Julián MC, Rubio V (1999). Design of a primer for ribosomal DNA internal transcribed spacer with enhanced specificity for ascomycetes. J Biotechnol.

[16] Hall M, Beiko RG. 16S rRNA gene analysis with QIIME2. Microbiome analysis: methods and protocols. 2018;:113–29. 10.1007/978-1-4939-8728-3_8 30298251

[17] Callahan BJ, McMurdie PJ, Rosen MJ, Han AW, Johnson AJA, Holmes SP (2016). DADA2: high-resolution sample inference from Illumina amplicon data. Nat Methods.

[18] Yang F-J. An implementation of naive bayes classifier. In: 2018 International conference on computational science and computational intelligence (CSCI). IEEE; 2018. p. 301–6.

[19] Abarenkov K, Nilsson RH, Larsson K-H, Alexander IJ, Eberhardt U, Erland S (2010). The UNITE database for molecular identification of fungi–recent updates and future perspectives. New Phytol.

[20] Stamatakis A (2014). RAxML version 8: a tool for phylogenetic analysis and post-analysis of large phylogenies. Bioinformatics.

[21] Callahan BJ, McMurdie PJ, Holmes SP (2017). Exact sequence variants should replace operational taxonomic units in marker-gene data analysis. ISME J.

[22] Maruyama H, Masago A, Nambu T, Mashimo C, Okinaga T (2020). Amplicon sequence variant-based oral microbiome analysis using QIIME 2. J Osaka Dent Univ.

[23] McMurdie PJ, Holmes S (2013). Phyloseq: an R package for reproducible interactive analysis and graphics of microbiome census data. PLoS ONE.

[24] Kumar S, Stecher G, Tamura K (2016). MEGA7: molecular evolutionary genetics analysis version 7.0 for bigger datasets. Mol Biol Evol.

[25] Myung IJ (2003). Tutorial on maximum likelihood estimation. J Math Psychol.

[26] Jukes TH, Cantor CR (1969). Evolution of protein molecules. Mammalian Protein Metabolism.

[27] Neath AA, Cavanaugh JE (2012). The bayesian information criterion: background, derivation, and applications. WIREs Comput Stats.

[28] Letunic I, Bork P (2007). Interactive tree of life (iTOL): an online tool for phylogenetic tree display and annotation. Bioinformatics.

[29] Rozas J, Ferrer-Mata A, Sánchez-DelBarrio JC, Guirao-Rico S, Librado P, Ramos-Onsins SE (2017). DnaSP 6: DNA sequence polymorphism analysis of large data sets. Mol Biol Evol.

[30] Leigh JW, Bryant D (2015). POPART: full-feature software for haplotype network construction. Methods Ecol Evol.

[31] Clement M, Snell Q, Walker P, Posada D, Crandall K. TCS: estimating gene genealogies. In: Parallel and Distributed Processing Symposium, International. IEEE Computer Society; 2002. p. 0184–0184.

[32] Jombart T (2008). Adegenet: a R package for the multivariate analysis of genetic markers. Bioinformatics.

[33] Jombart T, Balloux F, Dray S (2010). Adephylo: new tools for investigating the phylogenetic signal in biological traits. Bioinformatics.

[34] Huelsenbeck JP, Ronquist F, Nielsen R, Bollback JP (2001). Bayesian inference of phylogeny and its impact on Evolutionary Biology. Science.

[35] Drummond AJ, Rambaut A (2007). BEAST: bayesian evolutionary analysis by sampling trees. BMC Evol Biol.

[36] Fick SE, Hijmans RJ (2017). WorldClim 2: new 1-km spatial resolution climate surfaces for global land areas. Int J Climatol.

[37] Ihaka R, Gentleman R (1996). R: a language for data analysis and graphics. J Comput Graphical Stat.

[38] Royston P (1992). Approximating the Shapiro-Wilk W-test for non-normality. Stat Comput.

[39] Ruxton GD (2006). The unequal variance t-test is an underused alternative to Student’s t-test and the Mann–Whitney U test. Behav Ecol.

[40] Vargha A, Delaney HD (1998). The Kruskal-Wallis test and stochastic homogeneity. J Educational Behav Stat.

[41] Armstrong RA (2014). When to use the B onferroni correction. Ophthalmic Physiol Opt.

[42] Dray S, Legendre P, Peres-Neto PR (2006). Spatial modelling: a comprehensive framework for principal coordinate analysis of neighbour matrices (PCNM). Ecol Model.

[43] Anderson MJ, Walsh DC (2013). PERMANOVA, ANOSIM, and the Mantel test in the face of heterogeneous dispersions: what null hypothesis are you testing?. Ecol Monogr.

[44] Dixon P (2003). VEGAN, a package of R functions for community ecology. J Veg Sci.

[45] Kolde R, Kolde MR. Package ‘pheatmap.’ R package. 2018;1.

[46] Kassambara A, Kassambara MA. Package ‘ggpubr.’ R package version 01. 2020;6.

[47] Beals EW. Bray-Curtis ordination: an effective strategy for analysis of multivariate ecological data. Advances in ecological research. Elsevier; 1984. 1–55.

[48] Baroni TJ, Halling RE (2000). Some Entolomataceae (Agaricales) from Costa Rica. Brittonia.

[49] Swart WJ, Wingfield MJ (1991). Biology and Control of Sphaeropsis sapinea on Pinus species in South Mrica. Detail.

[50] Chuaseeharonnachai C, Somrithipol S, Suetrong S, Klaysuban A, Pornputtapong N, Jones EG (2017). Conioscypha Nakagirii, a new species from naturally submerged wood in Thailand based on morphological and molecular data. Mycoscience.

[51] Lombard L, Van der Merwe NA, Groenewald JZ, Crous PW (2015). Generic concepts in Nectriaceae. Stud Mycol.

[52] Tedersoo L, Bahram M, Põlme S, Kõljalg U, Yorou NS, Wijesundera R (2014). Global diversity and geography of soil fungi. Science.

[53] Nguyen NH, Song Z, Bates ST, Branco S, Tedersoo L, Menke J (2016). FUNGuild: an open annotation tool for parsing fungal community datasets by ecological guild. Fungal Ecol.

[54] Barr DJ (1980). An outline for the reclassification of the Chytridiales, and for a new order, the Spizellomycetales. Can J Bot.

[55] Jöreskog KG, Sörbom D (1982). Recent developments in structural equation modeling. J Mark Res.

[56] Rosseel Y (2012). Lavaan: an R package for structural equation modeling. J Stat Softw.

[57] Fernandes ÉK, Rangel DE, Braga GU, Roberts DW (2015). Tolerance of entomopathogenic fungi to ultraviolet radiation: a review on screening of strains and their formulation. Curr Genet.

[58] van den Brink J, de Vries RP (2011). Fungal enzyme sets for plant polysaccharide degradation. Appl Microbiol Biotechnol.

[59] Pullman GS, DeVay JE, Garber RH (1981). Soil solarization and thermal death: a logarithmic relationship between time and temperature for four soilborne plant pathogens. Phytopathology.

[60] Heinemeyer A, Fitter AH (2004). Impact of temperature on the arbuscular mycorrhizal (AM) symbiosis: growth responses of the host plant and its AM fungal partner. J Exp Bot.

[61] Pickles BJ, Egger KN, Massicotte HB, Green DS (2012). Ectomycorrhizas and climate change. Fungal Ecol.

[62] Duarte AG, Maherali H (2022). A meta-analysis of the effects of climate change on the mutualism between plants and arbuscular mycorrhizal fungi. Ecol Evol.

[63] Ruibal C, Platas G, Bills GF (2008). High diversity and morphological convergence among melanised fungi from rock formations in the Central Mountain System of Spain. Persoonia-Molecular Phylogeny and Evolution of Fungi.

[64] Bell AA, Wheeler MH (1986). Biosynthesis and functions of fungal melanins. Annu Rev Phytopathol.

[65] Jacobson ES (2000). Pathogenic roles for fungal melanins. Clin Microbiol Rev.

[66] Langfelder K, Streibel M, Jahn B, Haase G, Brakhage AA (2003). Biosynthesis of fungal melanins and their importance for human pathogenic fungi. Fungal Genet Biol.

[67] Vember VV, Zhdanova NN (2001). Peculiarities of linear growth of the melanin-containing fungi Cladosporium Sphaerospermum Penz. And Alternaria alternata (Fr.) Keissler. Mikrobiolohichnyi Zhurnal (Kiev Ukraine: 1993).

[68] Dadachova E, Casadevall A, Horikoshi K (2011). Melanin and resistance to Ionizing Radiation in Fungi. Extremophiles Handbook.

[69] Becker K, Stadler M (2021). Recent progress in biodiversity research on the Xylariales and their secondary metabolism. J Antibiot.

[70] Lodge DJ, Padamsee M, Matheny PB, Aime MC, Cantrell SA, Boertmann D (2014). Molecular phylogeny, morphology, pigment chemistry and ecology in Hygrophoraceae (Agaricales). Fungal Divers.

[71] Cedeño–Sanchez M. Three new species of Hypoxylon and new records of Xylariales from Panama. 2020.

